# Quantitative ultrasound radiomics guided adaptive neoadjuvant chemotherapy in breast cancer: early results from a randomized feasibility study

**DOI:** 10.3389/fonc.2024.1273437

**Published:** 2024-04-19

**Authors:** Archya Dasgupta, Daniel DiCenzo, Lakshmanan Sannachi, Sonal Gandhi, Rossana C. Pezo, Andrea Eisen, Ellen Warner, Frances C. Wright, Nicole Look-Hong, Ali Sadeghi-Naini, Belinda Curpen, Michael C. Kolios, Maureen Trudeau, Gregory J. Czarnota

**Affiliations:** ^1^ Department of Radiation Oncology, Sunnybrook Health Sciences Centre, Toronto, ON, Canada; ^2^ Department of Radiation Oncology, University of Toronto, Toronto, ON, Canada; ^3^ Physical Sciences, Sunnybrook Research Institute, Toronto, ON, Canada; ^4^ Division of Medical Oncology, Department of Medicine, Sunnybrook Health Sciences Centre, Toronto, ON, Canada; ^5^ Department of Medicine, University of Toronto, Toronto, ON, Canada; ^6^ Department of Surgical Oncology, Department of Surgery, Sunnybrook Health Sciences Centre, Toronto, ON, Canada; ^7^ Department of Surgery, University of Toronto, Toronto, ON, Canada; ^8^ Department of Medical Biophysics, University of Toronto, Toronto, ON, Canada; ^9^ Department of Electrical Engineering and Computer Sciences, Lassonde School of Engineering, York University, Toronto, ON, Canada; ^10^ Department of Medical Imaging, Sunnybrook Health Sciences Centre, Toronto, ON, Canada; ^11^ Department of Medical Imaging, University of Toronto, Toronto, ON, Canada; ^12^ Department of Physics, Ryerson University, Toronto, ON, Canada

**Keywords:** breast cancer, radiomics, quantitative ultrasound (QUS), neoadjuvant chemotherapy, adaptive chemotherapy, artificial intelligence

## Abstract

**Background:**

In patients with locally advanced breast cancer (LABC) receiving neoadjuvant chemotherapy (NAC), quantitative ultrasound (QUS) radiomics can predict final responses early within 4 of 16-18 weeks of treatment. The current study was planned to study the feasibility of a QUS-radiomics model-guided adaptive chemotherapy.

**Methods:**

The phase 2 open-label randomized controlled trial included patients with LABC planned for NAC. Patients were randomly allocated in 1:1 ratio to a standard arm or experimental arm stratified by hormonal receptor status. All patients were planned for standard anthracycline and taxane-based NAC as decided by their medical oncologist. Patients underwent QUS imaging using a clinical ultrasound device before the initiation of NAC and after the 1^st^ and 4^th^ weeks of treatment. A support vector machine-based radiomics model developed from an earlier cohort of patients was used to predict treatment response at the 4^th^ week of NAC. In the standard arm, patients continued to receive planned chemotherapy with the treating oncologists blinded to results. In the experimental arm, the QUS-based prediction was conveyed to the responsible oncologist, and any changes to the planned chemotherapy for predicted non-responders were made by the responsible oncologist. All patients underwent surgery following NAC, and the final response was evaluated based on histopathological examination.

**Results:**

Between June 2018 and July 2021, 60 patients were accrued in the study arm, with 28 patients in each arm available for final analysis. In patients without a change in chemotherapy regimen (53 of 56 patients total), the QUS-radiomics model at week 4 of NAC that was used demonstrated an accuracy of 97%, respectively, in predicting the final treatment response. Seven patients were predicted to be non-responders (observational arm (n=2), experimental arm (n=5)). Three of 5 non-responders in the experimental arm had chemotherapy regimens adapted with an early initiation of taxane therapy or chemotherapy intensification, or early surgery and ended up as responders on final evaluation.

**Conclusion:**

The study demonstrates the feasibility of QUS-radiomics adapted guided NAC for patients with breast cancer. The ability of a QUS-based model in the early prediction of treatment response was prospectively validated in the current study.

**Clinical trial registration:**

clinicaltrials.gov, ID NCT04050228.

## Introduction

Breast cancer is a common malignancy in women associated with significant morbidity and mortality (1). Clinical outcomes are determined predominantly by the stage of disease during diagnosis, its molecular characteristics, and treatment-related factors (2). Locally advanced breast cancer (LABC) denotes advanced disease of the primary site or regional lymph nodes and is associated with higher chances of recurrence and poorer outcomes than early breast cancer (3, 4). Neoadjuvant chemotherapy (NAC) is the standard of care for patients with LABC, resulting in down-staging disease, increasing operability aiding in breast conservation, and has shown survival benefits in specific molecular subtypes. Chemotherapy regimens for NAC are typically administered in a set manner over a period of months, and final treatment response is determined through histopathological evaluation a few weeks after completion of scheduled chemotherapy and surgery. Neoadjuvant treatment also enables patients to be stratified according to pathological response for adjuvant therapies.

Radiomics involves quantitative analysis of imaging data usually coupled with machine learning classifiers to arrive at a meaningful link to clinical endpoints (5, 6). Radiomic analyses can be undertaken on different morphological and functional imaging modalities like ultrasonography (US), computed tomography (CT), magnetic resonance imaging (MRI), and positron emission tomography (PET) (7). Quantitative ultrasound (QUS) carries out direct analysis of the raw radiofrequency (RF) data from ultrasound imaging devices, as opposed to standard B-mode US, which involves transformed data leading to loss of information (8–10). Quantitative ultrasonography relies on the elastic properties of the tissues, with analysis of different spectral parameters highlighting various microstructural properties like acoustic scatterer size, shape, density, and organization which can be related to cellular morphology and arrangement. With cancer-directed therapy like chemotherapy or radiation, microscopic tissue changes are expected to start immediately after the initiation of treatment. However, typically response is appreciated only after months into treatment resulting from cumulative cell death and tumor size changes, the latter which can then be appreciated through standard imaging modalities, which lack sensitivity to detect microstructural changes during early phases of therapy. Cell death resulting from chemotherapy or RT leads to events like cell fragmentation, pyknosis, and formation of apoptotic bodies and cell death structures leading to changes in scatterer elastic properties which can be effectively determined by QUS imaging as demonstrated from preclinical and clinical studies.

Radiomic analysis of QUS imaging in accurate determination of treatment response to chemotherapy and RT in breast and head-neck malignancies had been demonstrated in prospective observational studies (11–14). The current phase 2 randomized study was undertaken to study the feasibility of using QUS-based response prediction for adaptive chemotherapy in patients with breast cancer receiving NAC. This is the first clinical study using a radiomics-guided approach for individualized treatment in oncology.

## Methods

### Study design and participants

This prospective randomized phase 2 study was conducted at a single institute, Sunnybrook Health Sciences Centre, Toronto, Canada. The study was approved by the institutional ethics committee and registered with the clinicaltrials.gov registry (NCT04050228). Women older than 18 years with a histologic diagnosis of primary breast malignancy with size of primary tumor ≥ 5 cm longest-dimension without distant metastasis, or smaller tumor (>2 cm diameter) with bulky axillary nodes, and eligible for NAC (normal blood counts, creatinine, liver function tests, and cardiac function) were considered eligible for the study. Contraindications included inflammatory breast cancer, previous history of connective tissue disease, past history of dermatologic disease involving breast, Eastern Cooperative Oncology Group (ECOG) performance status ≥ 3 and known sensitivity to components in ultrasound gel. Written informed consent form was obtained from all the study participants.

Patients enrolled in the study were randomly assigned through 1:1 allocation using the block randomization method to observational arm or experimental arm (adaptive chemotherapy for predicted non-responders), with hormone receptor status as a stratification factor (positive or negative). Study participants and investigators were not blinded to the allocation arm. The funding agencies had no role in the study design, analysis, or interpretation of the results.

### Treatment procedures

Patients accrued in the study underwent QUS imaging before starting NAC (within 7 days) and after weeks 1 and 4 of NAC. QUS Data was acquired by experienced sonographers using a Sonix RP clinical system (Analogic Medical Corp.) with an L14-5/60 linear transducer (central frequency 6.5 MHz, bandwidth range 3.0-8.5 MHz) or GE LOGIC E9 system with ML6-15 linear transducer (central frequency 6.9 MHz, bandwidth range 4.5-9.9 MHz). The primary tumor was imaged at 1 cm intervals to encompass the entire span of the disease volumetrically. The region of interest (ROI) delineation corresponding to the tumor was carried out by the sonographers and individually verified by an expert breast radiologist and principal investigator. The raw radiofrequency data was extracted from the ROI. Then a fast Fourier transform (FFT)-based approach was applied, with data normalization carried out using a reference phantom approach, and various spectral parameters and texture features determined as described previously (10, 15). A QUS-radiomics model incorporating texture analysis based on a support vector machine-radial based function algorithm (SVM-RBF) developed from over 100 patient’s data was applied in order to monitor responses to chemotherapy for patients and classify them after 4 weeks of treatment as responders or non-responders (15).

Patients in the observation arm were planned for standard of care NAC with dose-dense AC-T or FEC-D regimens as decided by their treating medical oncologist. Typically, dose-dense AC-T chemotherapy consisted of doxorubicin 60 mg/m^2^ and cyclophosphamide 600 mg/m^2^ weekly (AC) for 4 cycles, followed by paclitaxel 175 mg/m^2^ every two weeks (T) for 4 cycles. FEC-D included 5-FU 500 mg/m^2^, epirubicin 100 mg/m^2^, and cyclophosphamide 500 mg/m^2^ 3 weekly (FEC) for 3 cycles followed by docetaxel 100 mg/m2 every 3 weeks for 3 cycles. Use of growth factors and monitoring of hemogram, liver function tests, and renal function tests were done as per standard institutional practice. QUS Imaging was carried out before starting and during NAC at different experimental times, as mentioned earlier, with the treating medical oncologists blinded to results, and no changes in scheduled chemotherapy regimens were made.

The experimental arm involved the start of NAC either with AC-T or FEC-D regimens as planned by oncologists (as for the observational arm). QUS-Radiomics model prediction results at 4 weeks were made available to patients’ medical oncologists and were used in conjunction with clinical findings to potentially adapt treatments. Any treatment alterations were decided by the responsible oncologist. The use of the radiomics model to classify patients as a responder or non-responder was carried out within 48 hours of week 4 QUS data acquisition. Treatment changes decided by treating medical oncologists typically involved an early switch to taxane regimens, using alternative chemotherapy regimens, or planning early surgery.

In both the arms, patients underwent surgery with mastectomy or breast conservation surgery as decided by the breast surgeon. All patients were treated with adjuvant radiation and further maintenance targeted therapy or endocrinal therapy as appropriate, according to standard institutional practice. Patients that had HER2+ status received trastuzumab treatment and patients without pathological complete response received capecitabine adjuvantly.

### Study outcomes

Being a phase 2 feasibility study, the primary objectives of the study included recruitment rate, refusal rates, the proportion of patients classified as non-responders, patient/physician acceptability of adaptive changes in response to QUS prediction, and proportion of patients randomized to experimental arm undergoing adaptive change to the chemotherapy arm. The recruitment rate was defined as the number of patients who underwent randomization divided by the study period (date of the last patient randomized – the date of the first patient randomized in months). The refusal rate was defined as 1 – (the number of patients who signed the informed consent for this study divided by the number of patients who were approached to enter this study). The proportion of patients classified as non-responders was defined as the number of patients classified as non-responders to their neoadjuvant chemotherapy by quantitative ultrasound divided by the number of patients randomized to the experimental arm. For response monitoring by QUS a score is determined mathematically which is a combination of calculated tumor QUS metrics for each patient individually. These are combined into a predictive score. Patients are classified as predicted responders if their predictive score is more than ** or as predicted non-responders if their QUS score is less than **. Response assessment was carried out based on histopathological evaluation by dedicated breast pathologists following surgery. For clinical response standard RECIST criteria are used based on tumor size initially using MRI (where available) or clinical assessment, or tumor size at the time of surgery. To be specific a modified RECIST score was used such that if on histopathology there was tumor chemotherapy response noted with remaining cellularity less than 1% patients were recognized to be responders. The few patients in this situation potentially had large radiological structures noted which on pathology were made of scarring from chemotherapy response with little to no viable cancer cells remaining on histopathology. This is consistent with previous work (12, 15, 16). Patient/physician acceptability rate: was defined as the number of patients who switched their chemotherapy regimen on the basis of quantitative ultrasound, divided by the number of patients who were classified as non-responders amongst those randomized to the experimental arm. The proportion of patients whose treatment was adaptively changed based on quantitative ultrasound was defined as the number of patients who had their neoadjuvant chemotherapy altered due to quantitative ultrasound divided by the number of patients randomized to the experimental arm.

### Statistical analysis

Since the current study was primarily designed as a phase 2 feasibility study, sample size calculation was based on convenience without formal statistical analysis. A total of 120 patients was decided with 60 patients allocated equally to the observational and experimental arms. After the accrual of half of the patients (60), an unplanned interim analysis was carried out, and reported here, since the rate of accrual was slowed down due to the ongoing COVID-19 pandemic. Descriptive analysis was performed to study the patient, disease, treatment-related factors and response rates. Image preprocessing, feature extraction, and radiomics model development were carried out using MATLAB R2016a (MathWorks). Other statistical tests were performed using IBM SPSS version 22 (IBM Corporation). Standard statistical methods were used to calculate test performance (16, 17) and computed in combined group patients (Observational Arm and Experimental Arm-Non-Adapted).

## Results

**Figure 1:**
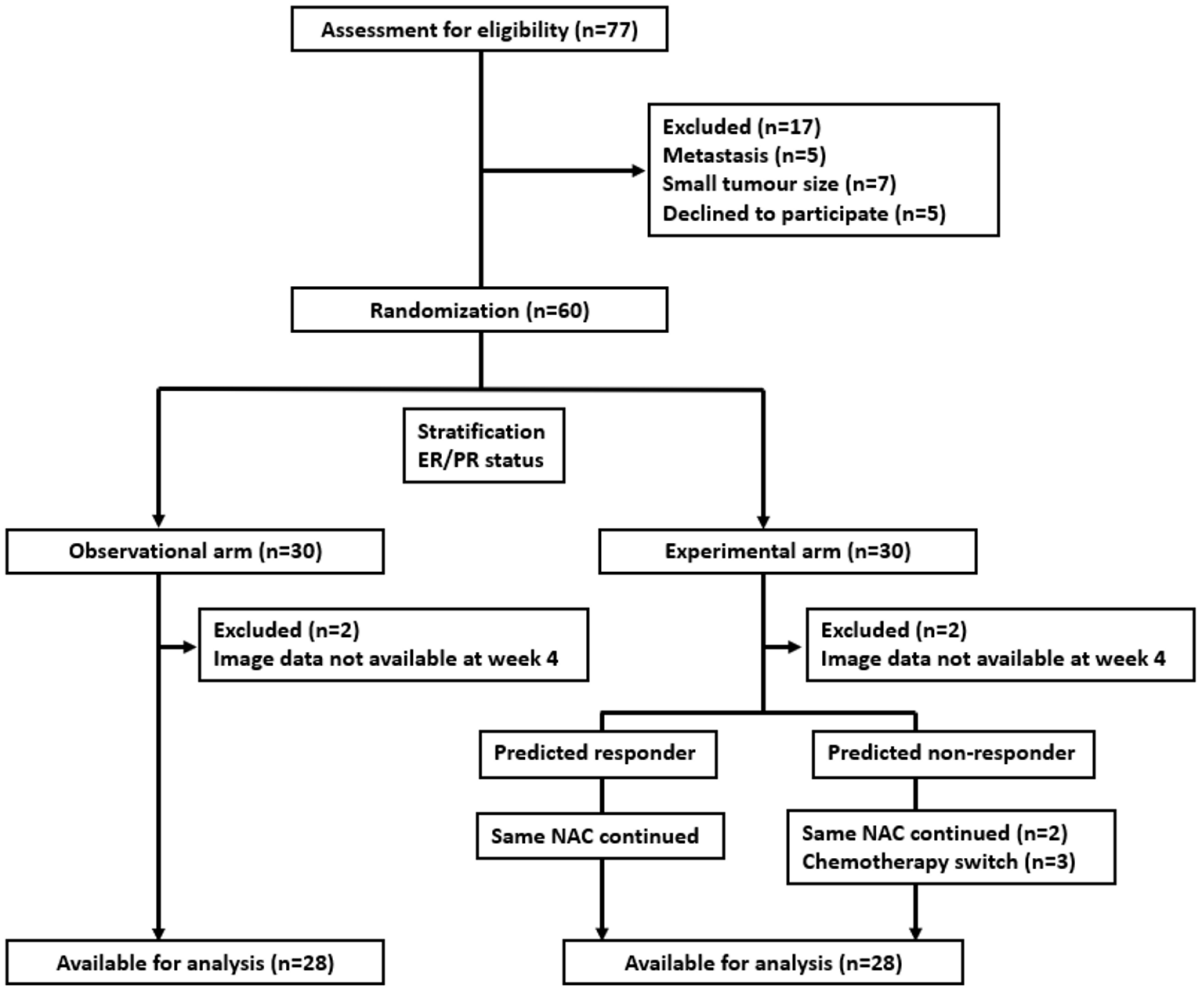
Consort diagram of the study.

**Figure 2:**
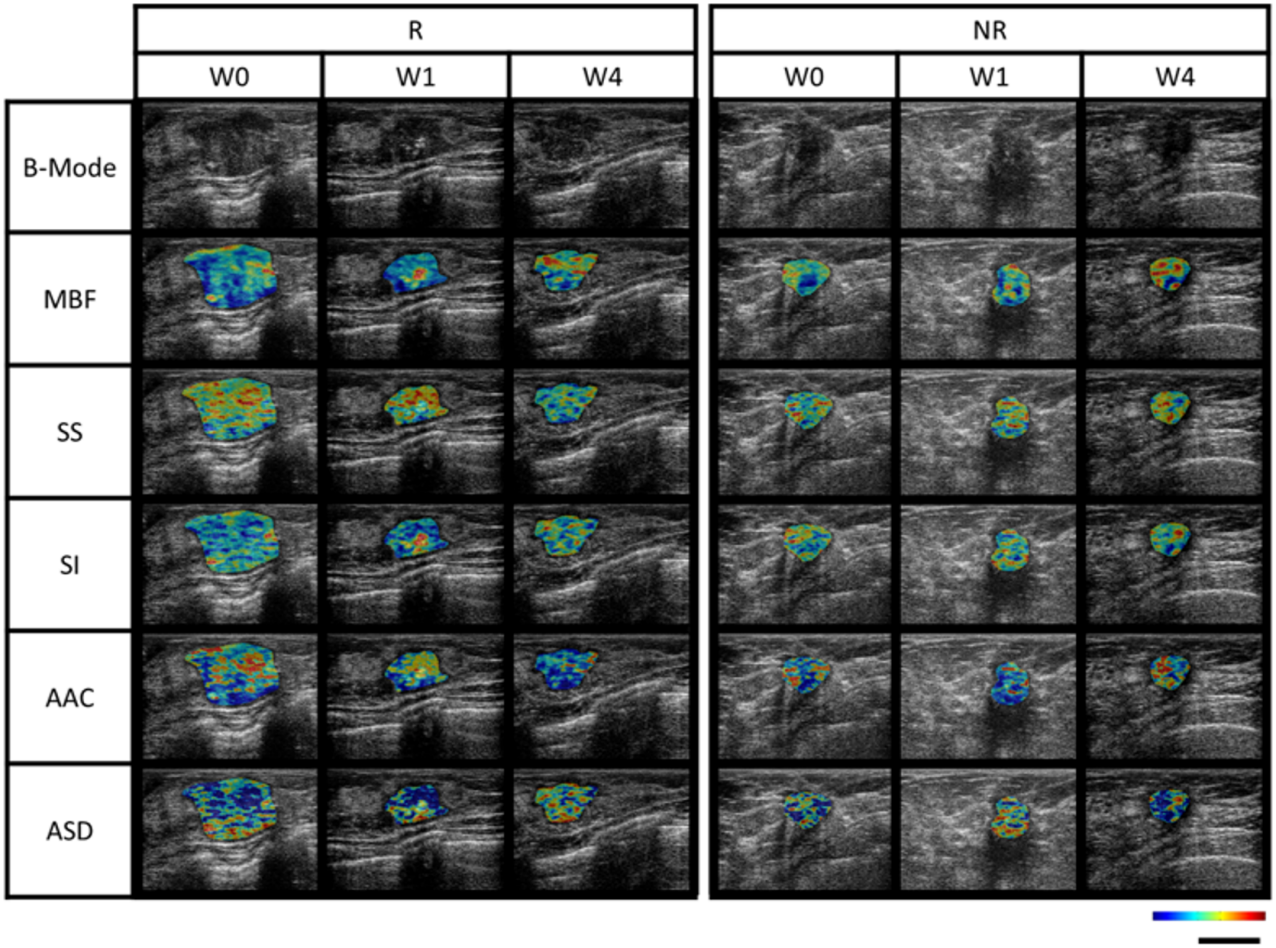
B-mode imaging and corresponding QUS-parametric maps at different time points (pretreatment or week 0, and week 1 and 4 of neoadjuvant chemotherapy) for 1 patient from responder and non-responder group. MBF range was from -9.6 dB to 34.0 dB, SS range was from -5.7 dB/MHz to 1.6 dB/MHz, SI range from -7.3 dB to 49.0 dB, AAC range was from 20.2 db/cm^3^ to 81.6 db/cm^3^, ASD range was from 40 μm to 171 μm. The scale bar represents a length of 2 cm. MBF, mid-band fit; SS, spectral slope; SI, spectral intercept; AAC, average acoustic concentration; ASD, average spectral diameter.

A total of 77 patients were screened for study eligibility between June 2018 and July 2021, with 60 accrued and randomized 1:1 with 30 patients each in both arms. In each arm, 2 patients were ineligible, resulting in 56 patients available for analysis, as presented in the consort diagram in **Figure 1**. Baseline features were comparable in both arms, as summarized in **Table 1**. The median age for patients in the observational arm and experimental arm was 49 years and 50 years, respectively. The median primary tumor size was 3.6 cm and 3.8 cm in the observational and experimental arms, respectively. The majority of patients (71%) received AC-T chemotherapy, while FEC-D was used in the remaining. Trastuzumab was used in 27% of the patients. B-mode Ultrasound images along with representative QUS parametric maps before treatment and after 1^st^ and 4^th^ week of NAC for one patient each from the responder and non-responder group are presented in **Figure 2**. Patient characteristics are presented in **Table 1** and **Supplementary Tables 1-3**. Specifically, amongst responders 19% were HR (hormonal receptor including ER/PR) +//Her2+, 47% were HR+Her2-, 8% HR-/Her2+ and 26% HR-/HER2-. Specifically, amongst non-responders 67% were HR+Her2-, and 33% HR-/HER2-. In our cohort, complete response rate was seen in 31%, partial response rate in 63%, and patients with stable or progressive disease made up 6% of all patients.

**Table 1:** Patient, disease, and treatment characteristics for patients in observational and experimental arm.

Characteristic	Observational Arm (n=28)	Experimental Arm (n=28)
Age (years)		
Median (Range)	49 (29-73)	50 (27-80)
Initial tumor size (cm)		
Median (Range)	3.6 (2.0-12.0)	3.8 (2.1-10.7)
Molecular Markers		
ER+	19	18
PR+	13	13
HER2+	7	8
TNBC	6	8
Histological Type		
IDC	26	25
IMC/Other	2	3
Chemotherapy		
AC-T	19	21
FEC-D	9	7
Trastuzumab	7	8
Treatment Response		
Responder	26	23 (27)
Non-Responder	2	5 (1)
**Response Rate**	93%	82% (96%)

With a total of 60 patients accrued over a period of 38 months, the recruitment rate was faster than anticipated (1.5 patients/month) but then affected by the COVID-19, else, the recruitment rate was >2.5 patients/month before the onset of the pandemic. The refusal rate for study participation was 8% mostly due to patients wishing to be allocated to the experimental arm. The proportions of non-responders were 7% and 18% in the observational arm and experimental arm, respectively.

**Figure 3:**
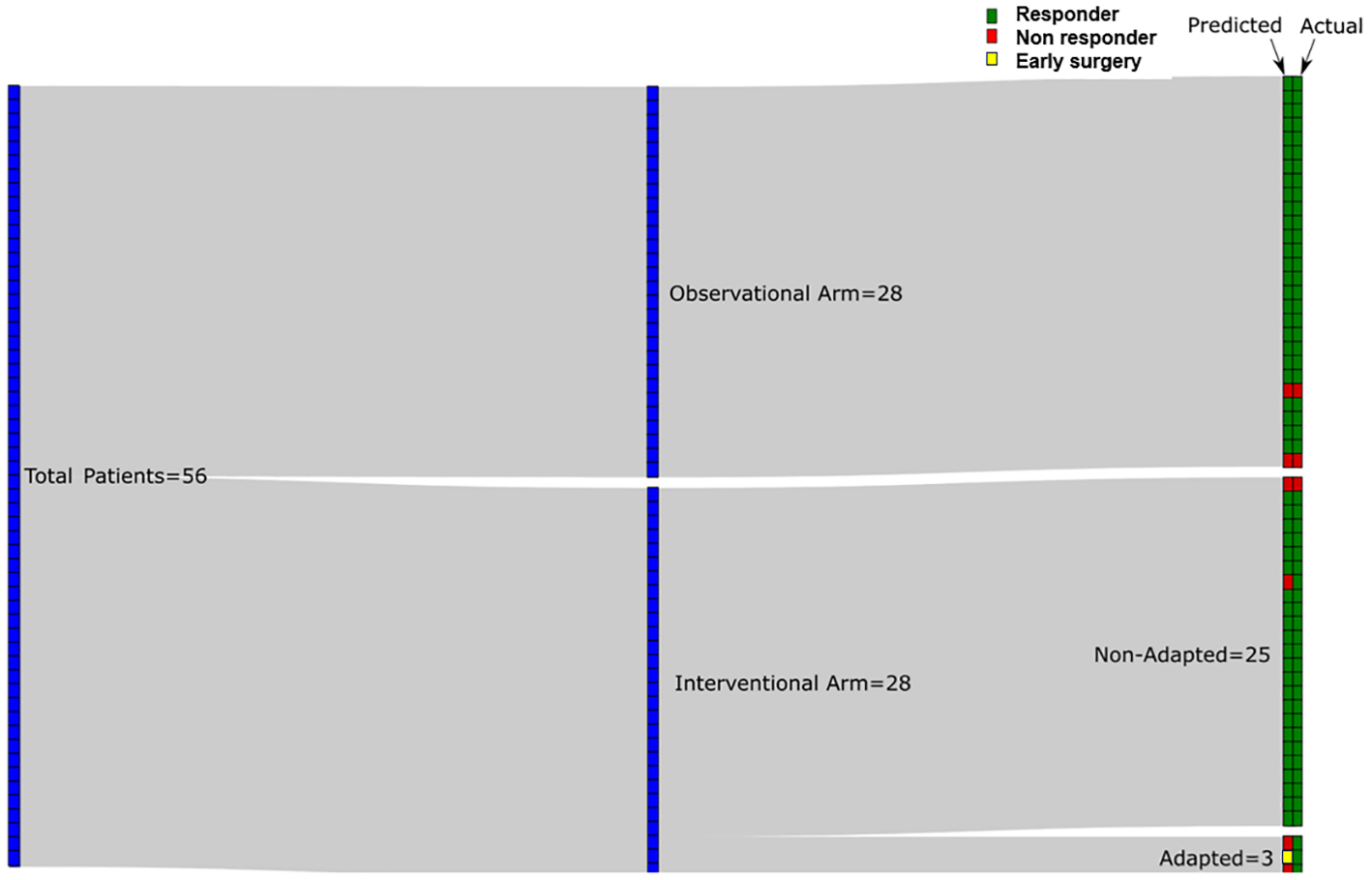
Sankey diagram for predicted response at week 4 using QUS-radiomics model with the final response on an individual patient basis. Red tiles indicate non-response (predicted or actual) and green tiles indicate response (predicted or actual). In the experimental arm 25/28 patients were not adapted based on information provided to their oncologist whereas 3/28 were adapted. Patient with early surgery is indicated with a yellow tile.

**Figure 4:**
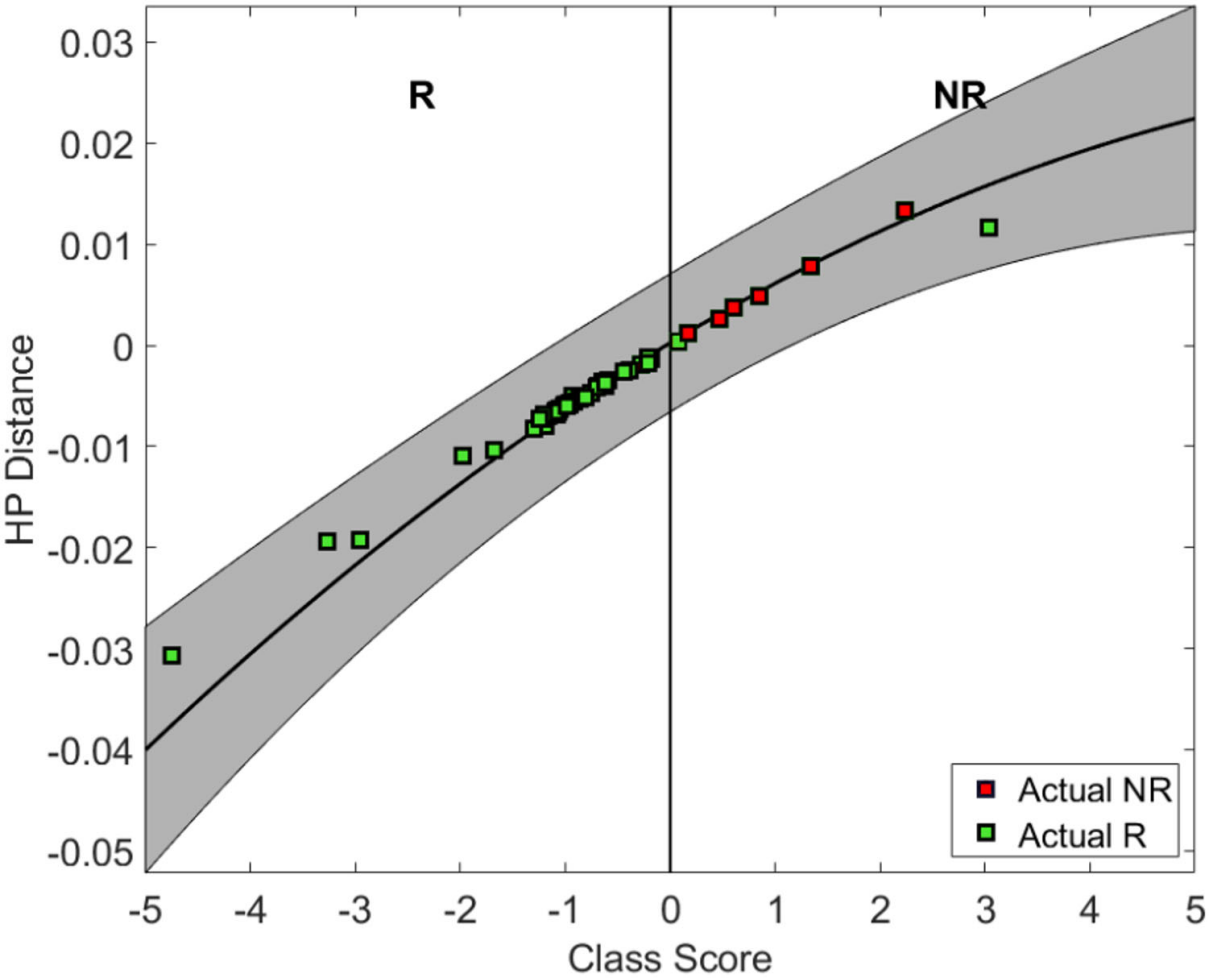
Individual patient predictions based on predictor class scores at week 4 for patient response. R indicates the zone (negative class score) for predicted response and NR indicates the zone (+ve class score) for non-response.

**Figure 5:**
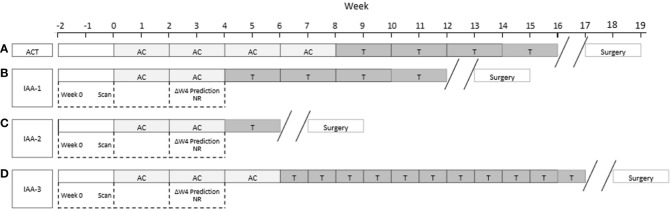
**(A)** Schematic diagram for the administration of standard AC-T chemotherapy. Weeks are shown from left to right. Typical durations are illustrated. **(B-D)** indicate the three patients in the Experimental Arm that were adapted (IAA-1, IIA-2, and IIA-3) in **Figure 3**. In **(B)** AC was shortened to move to T. In **(C)** the AC and T were shortened to move to surgery and in **(C)** AC was shortened and T was intensified,.

Using a QUS-radiomics prediction model at week 4 of NAC, a total of 7 patients were predicted to be non-responders based on surgical pathology (2 in the observational arm and 5 in the experimental arm). **Figures 3** and **4** demonstrate the prediction at 4 weeks on an individual patient basis with the final response across the two treatment arms. Of the 5 non-responders in the experimental arm, 3 patients (60%) were considered for adaptive chemotherapy based on physician decision and patient acceptance, whereas others (2 patients) continued on initial original planned NAC regimen. Changes made to chemotherapy are presented in **Figure 5**. All 2 predicted non-responders in the observational arm and 1 of 2 in the experimental arm (non-adapted branch) were true non-responders based on final evaluation following surgery. Considering patients who continued on an unchanged neoadjuvant regimen (Observation Arm and Experimental Arm Non-adapted), the sensitivity, specificity, and accuracy of the QUS-radiomics model at 4 weeks were 98%, 80%, and 97%, respectively (**Table 2**). All the three patients in whom adaptive chemotherapy was considered started with AC regimens, which were switched to taxane chemotherapy. Two of them continued receiving taxane (weekly taxane in one patient and dose-dense treatment in another patient), after which they were taken for surgery. The patient treated with weekly taxane also received trastuzumab. In the other patient, early surgery was considered after one cycle of taxane since the primary disease appeared to progress clinically.

**Table 2:** Classifier performance of QUS-radiomics model at week 4 of neoadjuvant chemotherapy.

Parameter	Value (95% confidence interval)
Sensitivity	98% (88-100%)
Specificity	80% (28-100%)
Positive predictive value	98% (89-99%)
Negative predictive value	80% (36-97%)
Accuracy	97% (88-99%)

Confidence intervals for sensitivity, specificity, and accuracy are "exact" Clopper-Pearson confidence intervals. Confidence intervals for predictive values are standard logit confidence intervals.

In the experimental non-adapted arm (n=25 of 28) there were 2 patients who were predicted non-responders and were actual non-responders at the end of their therapy. In the experimental adapted arm (n=3 of 28) there were 3 patients who were predicted non-responders but due to intervention ended up converting to responders. More specifically, for the three patients two had the first phase of their chemotherapy shortened and went on to the second phase of their chemotherapy (taxane) sooner and responded to that phase of chemotherapy. In one patient treatment was intensified in terms of frequency of taxane administration. An additional (third) patient had the second phase of their chemotherapy shortened due to a continued lack of response and went to surgery promptly removing all tumor - and was therefore considered a responder to salvage surgical treatment.

In the observational arm all patients predicted to be responders (n=26) and non-responders (n=2) ended up responding and not responding to their treatment, respectively (**Table 3**).

**Table 3:** Patient predicted and actual reponses to neoadjuvant chemotherapy.

Response		Study Arm		
Predicted	Actual	Observational	Experimental - Non-Adapted	Experimental - Adapted
Non-Responder	Non-Responder	2	1	0
Non-Responder	Responder	0	1	3
Responder	Responder	26	23	0
Responder	Non-Responder	0	0	0

## Discussion

Radiomics involving quantitative imaging analysis has led to promises in serving to generate prognostic and predictive biomarkers in oncology over the past decade (7, 17, 18). Imaging can aid in the non-invasive assessment of treatment response since histopathological or molecular characterization is often precluded during treatment due to the need for associated invasive procedures to obtain tissue and limited tissue sampling volumetrically (19, 20). Traditional morphological imaging can have limitations in determining response early in the course of treatment since the measurable effect of tumor size change is often manifested from cumulative cell death only after several weeks or months of treatment. Driven by encouraging results of QUS-radiomics in determining early responses in patients with breast cancer receiving NAC, the current phase 2 randomized controlled trial here was initiated to study the feasibility of adaptive chemotherapy for non-responders guided by QUS.

The current study prospectively validates the ability of QUS-radiomics to predict tumor response after 4 weeks of treatment during 4-5 months of NAC. Cell death starts immediately at the microscopic level within a few hours of initiating therapies in the form of chemotherapy or radiotherapy (21, 22). Quantitative ultrasound has been promising in predicting final treatment response as early as 24 hours of starting treatment for breast and head-neck malignancies. The cascade of events associated with cell death like nuclear fragmentation, pyknosis, or apoptosis leads to changes in tissue architecture and elastic properties, which can be detected by QUS. The results are better depicted when quantitative image analysis of QUS data is undertaken along with machine learning algorithms. In a multicenter study involving 59 patients with breast cancer, the sensitivity, specificity at week 4 of NAC in the prediction of final response was 80% and 82%, respectively (12). In a different study of 100 patients a better performing classifier was developed with an accuracy of 90% (15) which was used here. Better classification performance results using that classifier were obtained from the current study (98% accuracy). It is well known that the performance matrices of machine learning algorithms improve with an increase in the magnitude of data (patient number). The radiomics model used in the study was developed with QUS and QUS-texture features using an SVM-RBF classifier from 100 patients’ data, explaining the better results using the current model when applied to the patients in this study. These performance indices using QUS-radiomics provide robustness when used in a clinical setting early in the course of neoadjuvant therapy, where the window of possible treatment modifications can be considered based on individual patient responses. In recent work, it has been demonstrated that the inclusion of higher-order imaging features in the form of texture derivatives has further improved the performance of classifier models. Although such features were not included in the current model (study here initiated in 2018), the future inclusion of a larger number of patients (currently data available for >300 patients) and higher-order features will lead to further increased reliability of the model for future applications.

The classifier here was based on a response-monitoring model using a SVM-RBF algorithm to predict treatment responses. That algorithm has previously demonstrated cross-validated accuracies of 90% (sensitivity 90%, specificity 90%). The classifier used is based on 4 texture features from QUS parametric images generated from data at 4-weeks after the start of neoadjuvant chemotherapy from 100 patients (15) separate from those (n=56) in this study. The performance in the totally separate data set here was very good (accuracy 98%).

The response to NAC in patients with breast cancer can be varied, with the majority achieving partial response, approximately 15-40% having pathological complete response, and 20-30% have no significant response to the treatment. Identifying non-responders early in their course of therapy can provide an opportunity to either switch to a different chemotherapy regimen or early consideration of surgery rather than continuing an ineffective treatment for the next few months. The current work is the first study to use a QUS radiomics-guided approach for considering a treatment switch. Biological imaging like PET has been used to predict response early in NAC. In the AVATAXHER phase 2 randomized trial, fluorodeoxyglucose (FDG) PET was done before the second cycle of NAC for patients with HER 2 positive breast cancer (23). Predicted non-responders were randomly allocated to therapy intensification with the addition of bevacizumab or continuing the same regimen of docetaxel and trastuzumab-based treatment. The pathological complete response rates were higher (44% versus 24%) in patients receiving additional treatment with bevacizumab. In another study (PHERGain), chemotherapy de-escalation was considered for patients with HER 2 positive cancers having a positive response on FDG-PET after 2 cycles (24). Pathological complete response rate was seen in 38% of PET predicted responders receiving chemotherapy-free dual HER2 blockade with trastuzumab and pertuzumab, with survival data pending. Another approach using circulating tumor DNA (ctDNA) collected during the course of NAC has demonstrated a lack of ctDNA clearance to be a significant predictor of poor response and metastatic relapse (25). QUS can be used as a simple portable imaging modality in the clinic with excellent prediction accuracies (more than 90%), as demonstrated by the study here with the added advantage of lesser cost and lack of technical challenges associated with PET or liquid biopsy.

The current study was a randomized phase 2 trial primarily designed to assess the feasibility of QUS radiomics-guided adaptive chemotherapy approach and the acceptance of patients and physicians. Given the first step towards NAC modification, the change of chemotherapy was not considered mandatory for all predicted non-responders - QUS-based predictive information was provided to oncologists to incorporate into their practice according to their judgement. With prospective validation of the radiomics model in predicting non-responders, the robustness of the model has been established, which can be made for decision-making in future with a higher degree of confidence. In the current study, all the patients where switching chemotherapy was carried out in non-responders finally ended up being responders or had tumor removed sooner. In contrast, almost all patients in the observational arm or patients who were not adapted in the experimental arm, who were non-responders ended up as non-responders This suggests that the role of treatment escalation or switching therapies with consideration of more intense chemotherapy regimens might help improve response rates. It is important to note that the patient population reported here nevertheless is relatively small, and that treatment modification choices may benefit from identifying partial responders as well. Previous work has demonstrated the capability of QUS radiomics to be used for such a purpose.

It is important to emphasize the fact that it is meaningful to predict non-responders early before they are recognized clinically as non-responders. Relying only on clinical observations may result in loss of the opportunity to modify systemic treatment when ineffective to an effective different chemotherapy regimen. Proceeding to surgical management directly in such a scenario is also not ideal since it truncates systemic therapy which could potentially impact systemic relapse risk as well.

Pathological complete response matters at individual patient level given that it directs care and subsequent therapies, even if found not to be strong surrogate for event free survival or overall survival at trial level (26). For certain phenotypes of breast cancer (ER- PR- HER2-/HER2+), it is still very important to maximize pathological complete response as it not only prognosticates but predicts need for post operative therapies (27, 28). The protocol used here suggests that one can tailor a “personalized” approach to neoadjuvant therapy in order to maximize this individual patient benefit.

At present a QUS model is being developed to demarcate between pathological complete responders versus partial responder, which might have a more significant impact on outcomes, as a complete response has been shown to impact survival positively. Partial responders could also be considered for additional treatment with such a QUS tool when able to differentiate responders from partial responders (29). Future work will involve a phase 3 randomized controlled trial of chemotherapy intensification, where all predicted non-responders will be considered for more intensive chemotherapy.

## Conclusion

The current study was the first demonstration of the feasibility of QUS-radiomics guided adaptive neoadjuvant chemotherapy for patients with breast cancer, leading the way toward a phase 3 randomized controlled trial. The ability of QUS-radiomics model to predict non-responders was validated prospectively in this study with sensitivity, specificity, and accuracy of 98%, 80%, and 97%, respectively. Patients who were non-responders had their chemotherapy adapted based on QUS-radiomic monitoring of therapy response leading to improved response rates.

## Data availability statement

The raw data supporting the conclusions of this article will be made available by the authors, without undue reservation.

## Ethics statement

The studies involving humans were approved by Sunnybrook Health Sciences Centre Research Ethics Board. The studies were conducted in accordance with the local legislation and institutional requirements. The participants provided their written informed consent to participate in this study.

## Author contributions

AD: Data curation, Formal analysis, Investigation, Methodology, Writing – original draft, Writing – review & editing. DD: Formal analysis, Investigation, Methodology, Writing – review & editing, Software. LS: Data curation, Formal analysis, Investigation, Methodology, Writing – review & editing, Software. SG: Data curation, Investigation, Methodology, Writing – review & editing. RP: Data curation, Investigation, Methodology, Writing – review & editing. AE: Data curation, Investigation, Methodology, Writing – review & editing. EW: Data curation, Investigation, Methodology, Writing – review & editing. FW: Data curation, Investigation, Methodology, Writing – review & editing. NL-H: Data curation, Investigation, Methodology, Writing – review & editing. AS-N: Data curation, Investigation, Methodology, Writing – review & editing. BC: Data curation, Investigation, Methodology, Writing – review & editing. MK: Methodology, Writing – review & editing, Data curation, Investigation. MT: Data curation, Investigation, Methodology, Writing – review & editing. GC: Conceptualization, Data curation, Formal analysis, Funding acquisition, Investigation, Methodology, Project administration, Resources, Software, Supervision, Validation, Writing – original draft, Writing – review & editing.
