# Supplementary material for: Quantitative ultrasound radiomics guided adaptive neoadjuvant chemotherapy in breast cancer: early results from a randomized feasibility study
Source: Front Oncol. 2024 Apr 19;14:1273437. doi: 10.3389/fonc.2024.1273437 (PMC11066296; doi:10.3389/fonc.2024.1273437)
Supplement: Supplementary file 1 [file Table_1.docx]

**Supplementary Table 1: Observational Arm**

| Patient Number | Age | Size | Type | Grade | ER | PR | H2N | Chemo | Response (P/A) |
| --- | --- | --- | --- | --- | --- | --- | --- | --- | --- |
| O-1 | 37 | 4.4 | IDC | 2-3 | + | + | - | FECD | R/R |
| O-2 | 42 | 8.5 | IDC | 3 | + | - | - | ACT | R/R |
| O-3 | 67 | 4.0 | IDC | 2 | - | - | + | FECDH3 | R/R |
| O-4 | 60 | 2.4 | IDC | 2 | + | - | - | FECD | R/R |
| O-5 | 45 | 2.7 | IDC | 3 | - | - | + | ACTH4 | R/R |
| O-6 | 40 | 5.9 | IDC | 2 | + | + | - | FECD | R/R |
| O-7 | 56 | 3.5 | IDC | 3 | + | - | - | TC | R/R |
| O-8 | 41 | 7.5 | IDC | 2-3 | + | - | - | ACT | R/R |
| O-9 | 53 | 3.1 | IDC | 3 | - | - | - | ACT | R/R |
| O-10 | 63 | 7.4 | IDC | 2 | + | + | - | FECD | R/R |
| O-11 | 71 | 1.7 | IDC | 3 | + | - | + | TH | R/R |
| O-12 | 37 | 12.0 | IMC | 1-2 | + | + | - | FECD | R/R |
| O-13 | 32 | 2.6 | IDC | 2 | - | + | - | FECD | R/R |
| O-14 | 60 | 3.2 | IDC | 3 | - | - | - | ACT | R/R |
| O-15 | 72 | 3.4 | IDC | 3 | + | + | - | ACT | R/R |
| O-16 | 48 | 7.5 | IDC | 3 | + | - | + | FECDH3 | R/R |
| O-17 | 51 | 11.0 | IDC | 2 | + | + | - | FECD | R/R |
| O-18 | 73 | 7.8 | IDC | 3 | + | + | - | ACT | R/R |
| O-19 | 29 | 8.3 | IDC | 3 | + | + | - | ACT | R/R |
| O-20 | 43 | 2.4 | IDC | 3 | - | - | - | ACT | R/R |
| O-21 | 68 | 5.0 | IDC | 2 | + | + | - | ACT | R/R |
| O-22 | 51 | 3.3 | IDC | 3 | + | + | - | ACT | R/R |
| O-23 | 51 | 1.9 | IDC | 3 | - | - | - | ACT | NR/NR |
| O-24 | 41 | 4.8 | IDC | 3 | + | + | - | ACT | R/R |
| O-25 | 34 | 1.9 | IDC | 3 | - | - | - | TC | R/R |
| O-26 | 42 | 3.6 | IDC | 3 | - | - | + | ACTH4 | R/R |
| O-27 | 63 | 2.3 | IDC | 3 | - | - | - | ACT | R/R |
| O-28 | 34 | 3.2 | IDC | 2 | + | + | - | ACT | NR/NR |

IDC: invasive ductal carcinoma

ILC: invasive lobular carcinoma

IMC: invasive mammary carcinoma

O-#: observational patient number

EAN-#: experimental arm non-adapted patient

EAA-#: experimental arm adapted patient

FECD: 5-fluoruracil, epirubicin, cyclophosphamide - docetaxel (Taxotere)

ACT: adriamycin, cyclophosphamide - paclitaxel (Taxol)

TC: docetaxel (Taxotere), cyclophosphamide

TH: docetaxel (Taxotere), Herceptin

H: Herceptin

Numbers denote the number of cycles given for Herceptin**Supplementary Table 2: Interventional - Non Adapted Arm**

| Patient Number | Age | Size | Type | Grade | ER | PR | H2N | Chemo | Response (P/A) |
| --- | --- | --- | --- | --- | --- | --- | --- | --- | --- |
| EAN-1 | 47 | 3.0 | IDC | 3 | + | - | - | ACT | NR/NR |
| EAN-2 | 47 | 6.7 | IDC | 3 | + | - | - | ACT | R/R |
| EAN-3 | 50 | 1.9 | IDC | 3 | + | - | - | ACT | R/R |
| EAN-4 | 50 | 6.1 | IDC | 1-2 | + | + | - | FECD | R/R |
| EAN-5 | 35 | 5.5 | IDC | 2 | + | + | + | ACT/H4 | R/R |
| EAN-6 | 50 | 10.7 | IDC | 2 | + | + | + | ACT/H4 | R/R |
| EAN-7 | 51 | 4.0 | IDC | 3 | + | + | - | FECD | R/R |
| EAN-8 | 72 | 3.5 | IDC | 3 | + | + | + | FECD/H3 | NR/R |
| EAN-9 | 63 | 4.0 | IDC | 3 | - | - | - | ACT | R/R |
| EAN-10 | 49 | 4.9 | IDC | 2 | + | + | + | FECD/H3 | R/R |
| EAN-11 | 50 | 3.0 | IDC | 1 | + | + | - | ACT | R/R |
| EAN-12 | 64 | 3.4 | IDC | 1-2 | + | + | + | FECD/H3 | R/R |
| EAN-13 | 43 | 5.6 | IDC | 2-3 | - | - | - | ACT | R/R |
| EAN-14 | 80 | 10.0 | IDC | 3 | - | - | - | ACT | R/R |
| EAN-15 | 27 | 1.4 | IDC | 2-3 | - | - | - | ACT | R/R |
| EAN-16 | 45 | 9.5 | IDC | 3 | + | + | - | ACT | R/R |
| EAN-17 | 43 | 2.1 | IDC | 3 | - | - | - | ACT | R/R |
| EAN-18 | 53 | 2.5 | IDC | 3 | - | - | + | ACT/H4 | R/R |
| EAN-19 | 54 | 3.5 | IDC | 2 | + | - | + | FECD/H3 | R/R |
| EAN-20 | 68 | 3.6 | IDC | 3 | + | - | - | ACT | R/R |
| EAN-21 | 61 | 2.9 | IDC | 3 | - | - | - | FECD | R/R |
| EAN-22 | 44 | 6.4 | IDC | 3 | + | - | - | ACT | R/R |
| EAN-23 | 65 | 2.3 | IDC | 2 | - | - | - | ACT | R/R |
| EAN-24 | 32 | 2.9 | IDC | 2 | + | + | + | ACT/H4 | R/R |
| EAN-25 | 41 | 7.5 | IDC | 2 | + | + | - | ACT | R/R |

IDC: invasive ductal carcinoma

ILC: invasive lobular carcinoma

IMC: invasive mammary carcinoma

O-#: observational patient number

EAN-#:experimental arm non-adapted patient

EAA-#: experimental arm adapted patient

FECD: 5-fluoruracil, epirubicin, cyclophosphamide - docetaxel (Taxotere)

ACT: adriamycin, cyclophosphamide - paclitaxel (Taxol)

TC: docetaxel (Taxotere), cyclophosphamide

TH: docetaxel (Taxotere), Herceptin

H: Herceptin

Numbers denote the number of cycles given for Herceptin

**Supplementary Table 3: Experimental - Adapted Arm**

| Patient Number | Age | Size | Type | Grade | ER | PR | H2N | Chemo | Response (P/A) |
| --- | --- | --- | --- | --- | --- | --- | --- | --- | --- |
| EAA-1 | 60 | 7.3 | IDC | 2 | - | - | - | AC→T | NR→R |
| EAA-2 | 72 | 4.7 | IMC | 3 | - | - | - | ACT→surgery | NR→R* |
| EAA-3 | 31 | 8.8 | IDC | 3 | + | + | + | AC→wPH | NR→R |

IDC: invasive ductal carcinoma

ILC: invasive lobular carcinoma

IMC: invasive mammary carcinoma

O-#: observational patient number

EAN-#:interventional arm non-adapted patient

EAA-#: interventional arm adapted patient

FECD: 5-fluoruracil, epirubicin, cyclophosphamide - docetaxel (Taxotere)

ACT: adriamycin, cyclophosphamide - paclitaxel (Taxol)

TC: docetaxel (Taxotere), cyclophosphamide

TH: docetaxel (Taxotere), Herceptin

H: Herceptin

Numbers denote the number of cycles given for Herceptin

Patient EAA-1: AC→T - AC phase of chemotherapy was switched to T and patient had tumour response.

Patient EAA-2: ACT→surgery - AC phase of chemotherapy was switched to T and T was abandoned and patient had early surgery removing tumour

Patient EAA-3: AC→wTH - AC phase of chemotherapy was switched to weekly Taxol and patient had tumour response.
